# Genetic Variants in the p14ARF/MDM2/TP53 Pathway Are Associated with the Prognosis of Esophageal Squamous Cell Carcinoma Patients Treated with Radical Resection

**DOI:** 10.1371/journal.pone.0158613

**Published:** 2016-07-14

**Authors:** Jing Li, Yang Tang, Liu Huang, Qianqian Yu, Guangyuan Hu, Xianglin Yuan

**Affiliations:** Department of Oncology, Tongji Hospital, Tongji Medical College, Huazhong University of Science and Technology, Wuhan, Hubei province, China; Virginia Commonwealth University, UNITED STATES

## Abstract

The p14ARF/MDM2/ TP53 pathway is known to play an important role in tumor progression by cell cycle control, although the association between this pathway and the prognosis of esophageal squamous cell carcinoma (ESCC) is unclear. In this study, we explored the association between genetic variants in the p14ARF/MDM2/TP53 pathway and prognosis in ESCC patients with radical resection. 124 ESCC patients with radical resection were included in this retrospective study and genotyped using the MassArray method. According to multivariate Cox hazard analysis and multiple testing, the TC/CC genotype of *p14ARF* rs3814960 was shown to be strongly related to a decreased overall survival (OS) (HR = 2.77, 95% CI: 1.33–5.75, *P* = 0.006, *Pc* = 0.030) and disease-free survival (DFS) (HR = 2.45, 95% CI: 1.30–4.61, *P* = 0.005, *Pc* = 0.025). Moreover, patients with the DEL/A +AA genotype of *MDM2* rs34886328 had a notably increased OS (HR = 0.27, 95% CI: 0.13–0.56, *P* = 4.7×10^−4^, *Pc* = 0.003) and DFS (HR = 0.22, 95% CI: 0.11–0.43, *P* = 1.1×10^−5^, *Pc* = 6.6×10^−5^). We also found that these two SNPs had a cumulative effect on the prognosis of ESCC, with the OS (*P* < 0.001) and DFS (*P* < 0.001) being shortest for patients carrying both of these unfavorable genotypes. In conclusion, genetic variants of the p14ARF/MDM2/TP53 pathway are significantly related to OS and DFS, and may be predictors of the prognosis of ESCC after surgery. We speculate the individuals with the TC/CC genotype of *p14ARF* rs3814960 and/or the DEL/DEL genotype of *MDMD2* rs34886328 should have more aggressive treatment and may greatly benefit from early prediction and prevention of an unfavorable prognosis by genotyping before the initiation of therapy. These findings should be further validated in a larger population.

## Introduction

Esophageal cancer (EC) is the fourth most common cancer diagnosed in China [1], and squamous cell carcinomas account for more than 90% of esophageal cancer in high-risk areas such as north-central China, Central Asian countries, and Northern Iran [2]. Despite new developments in early diagnosis and treatment, including surgery, radiation and chemotherapy, prognosis remains poor owing to frequent local recurrence or distant metastasis [3,4]. The overall five-year survival rate of ESCC is still less than 15%, and the clinical variables currently used to predict outcomes are imprecise [5]. Thus, the identification of molecular prognostic markers might enable further risk stratification, which could be the first step towards the individualization of treatment strategies [6].

In addition to the patient- and treatment- related factors reported by previous studies, including tumor stage and chemoradiotherapy, plasma values of TP53 were recently found to be associated with the prognosis of ESCC [7]. Further, single nucleotide polymorphisms (SNPs) of *TP53* were found by us to be related to the development of ESCC previously [8]. *TP53* is a key regulator of the G1/S cell cycle checkpoint [9]. As an important negative regulator of TP53, MDM2 inhibits its function by concealing the activation domain of TP53 [10,11] and by promoting degradation of TP53, most likely through the ubiquitin-proteasome pathway [12,13]. p14ARF can activate the TP53 pathway by interacting with and inhibiting the ubiquitin ligase activity of MDM2, preventing the polyubiquitination, nuclear export, and cytoplasmic degradation of TP53 [14]. The p14ARF/MDM2/TP53 pathway is therefore critical for normal cell cycle progression [15], and abnormalities of the p14ARF/MDM2/TP53 pathway are important mechanisms in the development and progression of cancers [16].

*p14ARF*, *MDM2* and *TP53* have been shown to present frequent mutations in many tumors [17–19]; However, to the best of our knowledge, no studies have addressed the role of genetic variants of the p14ARF/MDM2/TP53 signaling pathway in the prognosis of ESCC. We postulated that SNPs in the p14ARF/MDM2/TP53 pathway may be associated with the survival and recurrence of ESCC. To verify this hypothesis, we selected six potentially functional SNPs from *p14ARF*, *MDM2* and *TP53* to discover their potential associations with the OS and DFS of ESCC patients treated with radical resection.

## Materials and Methods

### Patient population

We retrospectively analyzed 124 patients treated with esophagectomy at the Department of Thoracic Surgery, Tongji Hospital of Huazhong University of Science and Technology (Wuhan, Hubei Province, China) between March 2010 and December 2012. Patients who had R0 resection for proven ESCCs confirmed by pathologists were included. The exclusion criteria included perioperative death and distal metastasis and neoadjuvant treatments. Clinicopathological information was obtained retrospectively from patient records and authors had access to identifying information during and after data collection. The Am*erican Joint Committee on Cancer Staging Manual (7th edition*, *2010)* was used to assess the tumor stage. The Ethical Committee of Tongji Hospital, Tongji Medical College, Huazhong University of Science and Technology approved our study. Written informed consent was obtained from all individual participants included in the study. After surgery, patients were followed-up every 4 months for the first 2 years, and every 6 months thereafter. Clinical information and physical examinations were included in each follow-up visit. Routine diagnostic imaging methods were used, including barium meal fluoroscopy and computed tomography, as well as tumor marker assays. The last follow-up was in May 2015.

### Genotyping methods

We collected the patients’ paraffin sections after esophagectomy from the Department of Pathology, Tongji Hospital. Genomic DNA was extracted with QIAamp DNA FFPE tissue kits (56404; Qiagen, Dusseldorf, Germany) from paraffin sections. Haploview software was used to choose the single nucleotide polymorphisms (SNPs) in the p14ARF/MDM2/TP53 pathway. We chose three key genes (*p14ARF*, *MDM2* and *TP53*) along the p14ARF/MDM2/TP53 pathway by a candidate gene approach [20]. All of the SNPs had minor allele frequencies of greater than 20% in the Chinese population based on the HapMap HCB data, and all correlated alleles were captured at r^2^ > 0.8. We only selected SNPs that were located in the 5′ or 3′-UTR gene region, or were found to be associated with the risk of ESCC by our previous reports, for example rs1042522. SNPs in strong linkage disequilibrium with selected SNPs or SNPs that cannot be determined in a well were excluded. Six SNPs were finally selected. For all six SNPs, genotypes were determined using the MassArray system (Sequenom iPLEX assay, San Diego, CA). The sample DNA was amplified by a multiplex PCR reaction, and the PCR products were then used for a locus-specific single-base extension reaction. Finally, the resulting products were desalted and transferred to a 384-element SpectroCHIP array. The alleles were discriminated by mass spectrometry (Sequenom).

### Statistical analysis

The end points for this study were OS and DFS. OS was defined as the length of time (in months) from the date of surgery to the last follow-up, or death from any cause. DFS was defined as the time (in months) from surgery to the occurrence of tumor relapse. SPSS 16.0 statistical software (SPSS Inc., Chicago, IL) was used for the statistical analysis. Patients were divided into groups according to their genotypes, and Cox proportional hazard analysis was applied to estimate the hazard ratio (HR) and 95% confidence intervals (CIs) of all possible prognostic factors. Moreover, multivariate Cox regression analysis was used for the adjustment of covariates. The influences of the genotypes on the prognosis were assessed by Kaplan–Meier analysis and compared with log-rank tests. For genotype analysis, *P*-values were corrected by the Benjamini and Hochberg False Discovery Rate correction.

## Results

### Patient characteristics and prognosis

A total of 124 patients were included in this study, consisting of 96 males and 28 females. Their characteristics are listed in Table 1. The median age of all patients was 58 years (range: 40–76 years); 61.3% smoked tobacco, 54.0% drank alcohol, 16.9% had stage III disease, and 42 (33.9%) received adjuvant therapy.

**Table 1:** Patient-, tumor- and therapy-related characteristics and their association with OS and DFS in ESCC patients (*n* = 124).

Characteristic		No. of patients	OS				DFS			
			Univariate analysis		Multivariate analysis		Univariate analysis		Multivariate analysis	
			Hazard ratio (95% CI)	*P*	Hazard ratio (95% CI)	*P*	Hazard ratio (95% CI)	*P*	Hazard ratio (95% CI)	*P*
Gender	Male	96 (77.4%)	1		1		1		1	
	Female	28 (22.6%)	0.43 (0.17–1.10)	0.078	0.45 (0.14–1.50)	0.196	0.57 (0.27–1.22)	0.151	0.53 (0.19–1.43)	0.208
Age, years	<58	57 (46.0%)	1		1		1		1	
	≥58	67 (54.0%)	0.70 (0.38–1.30)	0.257	0.88 (0.44–1.75)	0.706	0.54 (0.31–0.95)	0.031	0.69 (0.37–1.27)	0.234
Tobacco smoking	Never	48 (38.7%)	1		1		1		1	
	Ever	76 (61.3%)	1.33 (0.70–2.55)	0.387	1.05 (0.49–2.26)	0.906	1.34 (0.75–2.41)	0.326	1.25 (0.61–2.57)	0.541
Alcohol drinking	Never	57 (46.0%)	1		1		1		1	
	Ever	67 (54.0%)	1.29 (0.69–2.40)	0.429	0.82 (0.40–1.68)	0.592	1.02 (0.59–1.78)	0.938	0.61 (0.32–1.16)	0.131
Tumor length, cm	≤3	52 (41.9%)	1		1		1		1	
	>3	72 (58.1%)	1.21 (0.64–2.29)	0.557	0.83 (0.42–1.64)	0.583	1.34 (0.75–2.39)	0.318	1.02 (0.54–1.93)	0.942
Tumor location	Upper	15 (12.1%)	1		1		1		1	
	Middle	54 (43.5%)	1.08 (0.36–3.23)	0.892	1.13 (0.36–3.55)	0.829	1.01 (0.41–2.49)	0.983	0.91 (0.35–2.37)	0.848
	Lower	55 (44.4%)	1.53 (0.52–4.45)	0.438	1.90 (0.60–6.02)	0.277	1.02 (0.41–2.51)	0.971	0.90 (0.32–2.53)	0.844
Differential degree	Well	73 (58.9%)	1		1		1		1	
	Middle	46 (37.1%)	1.45 (0.77–2.72)	0.249	1.27 (0.65–2.46)	0.488	1.33 (0.75–2.35)	0.333	1.17 (0.64–2.14)	0.608
	Poor	5 (4.0%)	1.63 (0.38–6.95)	0.510	0.89 (0.18–4.32)	0.885	1.28 (0.30–5.36)	0.741	1.28 (0.27–6.11)	0.757
Tumor stage ^a^	Ⅰ	49 (39.5%)	1		1		1		1	
	Ⅱ	54 (43.5%)	2.11 (0.95–4.66)	0.066	2.90 (1.19–7.09)	**0.020**	1.63 (0.82–3.26)	0.166	1.76 (0.81–3.83)	0.156
	Ⅲ	21 (16.9%)	3.98 (1.70–9.32)	**0.001**	4.00 (1.59–10.05)	**0.003**	4.25 (2.03–8.89)	**<0.001**	3.88 (1.72–8.76)	**0.001**
Adjuvant therapy	No	82 (66.1%)	1		-		1		-	
	Yes	42 (33.9%)	2.83 (1.52–5.24)	**0.001**			2.97 (1.70–5.20)	**<0.001**		

OS, overall survival; DFS, disease-free survival; ESCC, esophageal squamous cell carcinoma; HR, hazard ratio; CI, confidence interval

Note: Multivariate analyses in this table were adjusted for patient sex, age, smoking status, drinking status, tumor length, tumor location, differential degree and tumor stage. Adjuvant therapy was not included in the multivariable analysis, because this depends on the tumor stage.

At a median follow-up interval of 37 months (range: 3–61 months), a total of 41 (33.1%) patients had died and 50 (40.3%) patients had developed tumor recurrences. The median OS was 37 months, and the median DFS was 35 months. While in patients who had developed tumor recurrences, the median OS was 20 months, and the median DFS was 13.5 months. Possible associations between patient-, tumor-, and therapy-related characteristics and prognosis, tested by univariate and multivariate analyses (Table 1), revealed that patients with stage III disease had decreased OS (HR = 4.00, 95% CI: 1.59–10.05, *P* = 0.003) and DFS (HR = 3.88, 95% CI: 1.72–8.76, *P* = 0.001) of ESCC. Patients who underwent adjuvant therapy had decreased OS and DFS compared with their counterparts who did not (HR = 2.83, 95% CI: 1.52–5.24, *P* = 0.001; HR = 2.97, 95% CI: 1.70–5.20, *P* < 0.001, respectively). Tumor length, tumor location, and differential degree were not associated with OS or DFS in this population.

### Effects of single SNPs in the p14ARF, MDM2 and TP53 genes on prognosis

**Fig 1:**
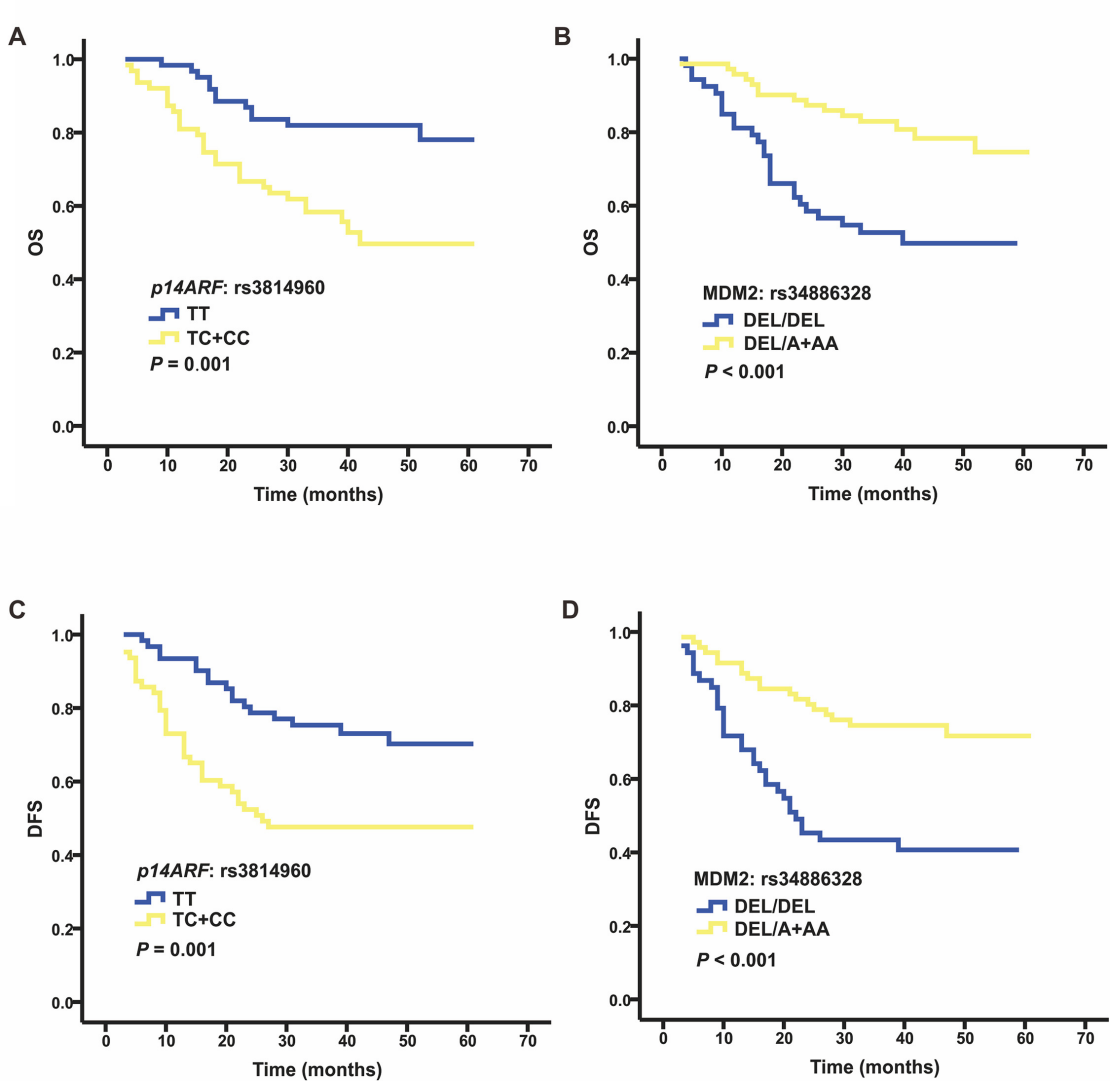
Prognostic value of each genotype in esophageal squamous cell carcinoma. Kaplan–Meier curves of overall survival (OS) for all patients for (A) *p14ARF* rs3814960 and (B) *MDM2* rs34886328. Kaplan–Meier curves of disease-free survival (DFS) for all patients for (C) *p14ARF* rs3814960 and (D) *MDM2* rs34886328.

Six candidate SNPs in the p14ARF/MDM2/TP53 pathway were screened by MassArray in 124 patients for the prognosis value (Table 2). The frequencies of the polymorphisms used in this study and in the general Chinese population are listed in S1 Table. We found that two SNPs (*p14ARF* rs3814960 and *MDM2* rs34886328) were significantly associated with the prognosis of ESCC (Fig 1), determined by the Kaplan–Meier method. Patients with the TC/CC genotype of *p14ARF* rs3814960 had significantly decreased OS (*P* = 0.001) and DFS (*P* = 0.001), while patients with the DEL/A +AA genotype of *MDM2* rs34886328 had significantly increased OS (*P* < 0.001) and DFS (*P* < 0.001). The other four SNPs were not found to be related to the OS or DFS of ESCC by the Kaplan–Meier method.

**Table 2:** Genes and single-nucleotide polymorphisms selected for analysis.

Genes and single-nucleotide polymorphisms	Allelic change	Functional consequence
*p14AR*F		
rs3814960	T/C	5 prime UTR variant
*MDM2*		
rs34886328	DEL/A	3 prime UTR variant
rs1632248	C/T	3 prime UTR variant
rs937283	A/G	5 prime UTR variant
*TP53*		
rs1050541	T/G	3 prime UTR variant
rs1042522	C/G	Missense variant

Furthermore, multiple Cox proportional hazard analyses with adjustments for patient sex, age, smoking status, drinking status, tumor length, tumor location, differential degree and tumor stage revealed that the TC/CC genotype of *p14ARF* rs3814960 was strongly related to a decreased OS (HR = 2.77, 95% CI: 1.33–5.75, *P* = 0.006, *Pc* = 0.030) and DFS (HR = 2.45, 95% CI: 1.30–4.61, *P* = 0.005, *Pc* = 0.025). Moreover, patients with the DEL/A +AA genotype of *MDM2* rs34886328 had a notably increased OS (HR = 0.27, 95% CI: 0.13–0.56, *P* = 4.7×10^−4^, *Pc* = 0.003) and DFS (HR = 0.22, 95% CI: 0.11–0.43, *P* = 1.1×10^−5^, *Pc* = 6.6×10^−5^) (Table 3, S2–S7 Tables). Similar analyses of the other four SNPs showed no associations between any genotype and OS or DFS.

**Table 3:** Associations between genotypes of single SNPs and prognosis values of ESCC.

Polymorphism and Genotype	No. of patients	OS					DFS				
		Univariate analysis		Multivariate analysis			Univariate analysis		Multivariate analysis		
		Hazard ratio (95% CI)	*P*	Hazard ratio (95% CI)	*P*	*P*_c_	Hazard ratio (95% CI)	*P*	Hazard ratio (95% CI)	*P*	*P*_c_
*p14AR*F: rs3814960											
TT	61	1		1			1		1		
TC+CC	63	3.00 (1.52–5.90)	0.002	2.77 (1.33–5.75)	0.006	0.030	2.51 (1.39–4.52)	0.002	2.45 (1.30–4.61)	0.005	0.025
*MDM2*: rs34886328											
DEL/DEL	53	1		1			1		1		
DEL/A +AA	71	0.34 (0.18–0.64)	0.001	0.27 (0.13–0.56)	4.7×10^−4^	0.003	0.35 (0.20–0.61)	2.9×10^−4^	0.22 (0.11–0.43)	1.1×10^−5^	6.6×10^−5^
*MDM2*: rs1632248											
CC	86	1		1			1		1		
CT+TT	38	0.85 (0.43–1.67)	0.635	0.74 (0.36–1.53)	0.418	-	0.83 (0.45–1.53)	0.544	0.79 (0.41–1.51)	0.473	-
*MDM2*: rs937283											
AA	77	1		1			1		1		
AG+GG	47	1.09 (0.58–2.04)	0.790	1.29 (0.65–2.58)	0.470	-	0.91 (0.51–1.62)	0.740	0.829 (0.45–1.54)	0.553	-
*TP53*: rs1050541											
TT	48	1		1			1		1		
TG+ GG	76	0.89 (0.48–1.65)	0.699	0.78 (0.41–1.50)	0.459	-	1.12 (0.63–2.00)	0.693	0.99 (0.54–1.80)	0.973	-
*TP53*: rs1042522											
CC	36	1		1			1		1		
CG+ GG	88	1.11 (0.56–2.21)	0.770	0.98 (0.47–2.04)	0.962	-	1.18 (0.63–2.22)	0.606	1.033 (0.53–2.00)	0.922	-

OS, overall survival; DFS, disease-free survival; ESCC, esophageal squamous cell carcinoma; HR, hazard ratio; CI, confidence interval; DEL, deletion

Note: Multivariate analyses in this table were adjusted for patient sex, age, smoking status, drinking status, tumor length, tumor location, differential degree and tumor stage. Adjuvant therapy was not included in the multivariable analysis, because this depends on the tumor stage. *P*c: *P*- value corrected by Benjamini and Hochberg False Discovery Rate correction.

### Combined effect of SNPs on prognosis

For these analyses, we defined the TC/CC genotype of *p14ARF* rs3814960 and the DEL/DEL genotype of *MDMD2* rs34886328 as “unfavorable” genotypes, as these genotypes were associated with worse prognosis. We grouped patients according to their number of unfavorable genotypes (i.e. 0, 1 or 2). Multivariate Cox proportional hazard analyses confirmed that for OS, the HR for individuals with one unfavorable genotype was 4.24 (95% CI: 1.34–13.41, *P* = 0.014; Table 4, S8 Table), and the HR for those with both unfavorable genotypes was 12.62 (95% CI: 3.69–43.14, *P* < 0.001). Cox proportional hazard analyses showed that for DFS, the HR for individuals with one unfavorable genotype was 2.94 (95% CI: 1.17–7.41, *P* = 0.022), and the HR for those with both unfavorable genotypes was 13.09 (95% CI: 4.73–36.24, *P* < 0.001). The Strengthening the Reporting of Observational Studies in Epidemiology (STROBE) Statement is listed in S9 Table and the raw clinical data is listed in S10 Table.

**Table 4:** Associations between genotypes and prognosis values of ESCC (combined).

No. of unfavorable Genetypes	No. of patients	OS				DFS			
		Univariate analysis		Multivariate analysis		Univariate analysis		Multivariate analysis	
		Hazard ratio (95% CI)	*P*	Hazard ratio (95% CI)	*P*	Hazard ratio (95% CI)	*P*	Hazard ratio (95% CI)	*P*
0	35	1		1		1		1	
1	62	3.30 (1.12–9.75)	0.031	4.24 (1.34–13.41)	0.014	2.07 (0.88–4.85)	0.095	2.94 (1.17–7.41)	0.022
2	27	10.08 (3.37–30.16)	<0.001	12.62 (3.69–43.14)	<0.001	7.72 (3.24–18.38)	<0.001	13.09 (4.73–36.24)	<0.001

OS, overall survival; DFS, disease-free survival; ESCC, esophageal squamous cell carcinoma; HR, hazard ratio; CI, confidence interval

Note: Multivariate analyses in this table were adjusted for patient sex, age, smoking status, drinking status, tumor length, tumor location, differential degree and tumor stage. Adjuvant therapy was not included in the multivariable analysis, because this depends on the tumor stage.

## Discussion

The current study evaluated genetic variants of *p14ARF*, *MDM2* and *TP53* to discover their potential associations with the prognosis of ESCC patients who underwent radical resection. Among them, two SNPs, *p14ARF* rs3814960 and *MDM2* rs34886328, were found to be significantly associated with the OS and the DFS of ESCC. The TC/CC genotype of *p14ARF* rs3814960 was strongly related to a decreased OS and DFS. Moreover, patients with the DEL/A +AA genotype of *MDM2* rs34886328 had a notably increased OS and DFS. Furthermore, we found that these SNPs had a cumulative effect on the prognosis of ESCC, with the OS and DFS being shortest for patients carrying both of these unfavorable genotypes. To the best of our knowledge, this is the first study to address the associations between genetic variants in the p14ARF/MDM2/TP53 pathway and the prognosis of ESCC, and it is the first study to address the associations between rs34886328, rs1632248, and ESCC.

The progression of cancer is a complex process involving environmental, genetic, treatment-related and other factors. The mechanisms of the relevance of the p14ARF/MDM2/TP53 pathway to the prognosis of ESCC is not clear. The failure of cell cycle control is a fundamental step in ESCC onset and progression. Key regulation of the G1/S cell cycle checkpoint is provided by TP53, together with MDM2 and p14ARF. The N-terminus of MDM2 binds to the transactivation domain of TP53 and inhibits its transcriptional activity, and MDM2 also regulates the TP53 protein level. TP53 is targeted for nuclear export and degradation in the cytoplasm through the ubiquitin-proteasome system, where MDM2 functions as an E3 ubiquitin ligase [21,22]. An autoregulatory loop occurs between TP53 and MDM2, where high TP53 transactivation is opposed by MDM2 upregulation [23]. p14ARF also takes part in this loop, and is located in the nucleolus, where it sequesters MDM2, inactivates it, and allows stabilization of TP53 [24]. This is regulated by TP53 in an autoregulatory feedback loop [25]. Generally speaking, the above evidence suggests that the p14ARF/MDM2/TP53 pathway is a vital regulator in tumor development and progression, indicating the biological plausibility of the relevance of the p14ARF/MDM2/TP53 pathway to the prognosis of ESCC, as shown by our research.

Indeed, complexities of cellular signaling pathways often means that a single SNP may produce a modest or undetectable effect, whereas the amplified effects of combined SNPs in the same pathway may enhance predictive power. When we combined two SNPs in different genes, both showing significant association with the prognosis of ESCC, we found substantial worse OS and DFS for patients with two unfavorable genotypes compared with those with no unfavorable genotypes. These results suggest that multiple genetic variants within the p14ARF/MDM2/TP53 pathway have a cumulative influence and may further enhance predictive power.

Our study suggests that the *p14ARF* rs3814960 and *MDM2* rs34886328 SNPs can be used as predictive biomarkers of ESCC after radical resection in genotyping assays, in addition to the tumor stage. Thus, we speculate the individuals with the TC/CC genotype of *p14ARF* rs3814960 and/or the DEL/DEL genotype of *MDMD2* rs34886328 should have more aggressive treatment and may greatly benefit from early prediction and prevention of an unfavorable prognosis by genotyping before the initiation of therapy. Moreover, our findings suggest a possible role of the p14ARF/MDM2/TP53 pathway in the progression of ESCC, which will aid in the discovery of targets to treat ESCC in future research.

In addition, in the present study we analyzed the prognostic role of genetic variants of the p14ARF/MDM2/TP53 pathway in ESCC patients who had not received neoadjuvant therapy mainly because chemotherapy and/or radiation may have an impact on the genetic variants in the tumor tissue. Thus, only 16.9% patients had stage III disease and only 40.3% patients had developed tumor recurrences in our study.

However, because of the retrospective nature of this study, and the relatively small population, our findings need to be confirmed by larger, multicenter, prospective studies. Due to the substantial ethnic variation in SNP frequencies, our results, which were demonstrated in a Han Chinese population, should be validated in different ethnic backgrounds. Moreover, rs3814960 and rs34886328 warrant further investigation to identify the causative SNPs and their molecular mechanisms. Furthermore, it is well-known that the p14ARF/MDM2/TP53 pathway is one of the most important signaling pathways in cancer development, so we need to explore the potential role of this pathway in the progression of ESCC, which could provide novel insight into the treatment of ESCC. We will also carry out further research to discover the impact of SNPs in the p14ARF/MDM2/TP53 pathway on cancer progression in our cohort.

## Conclusions

This is the first study to evaluate the associations between genetic variations in tumor tissue in the p14ARF/MDM2/TP53 pathway and the prognosis of ESCC. Two SNPs in the p14ARF/MDM2/TP53 pathway, *p14ARF* rs3814960 and *MDM2* rs34886328, were found to be significantly associated with the OS and DFS of ESCC, and may thus serve as predictors of ESCC, if these results are further validated in a larger population. Thus, we speculate the individuals with the TC/CC genotype of *p14ARF* rs3814960 and/or the DEL/DEL genotype of *MDMD2* rs34886328 should have more aggressive treatment and may greatly benefit from early prediction and prevention of an unfavorable prognosis by genotyping before the initiation of therapy.

## Supporting Information

S1 Table The frequencies of the polymorphisms used in this study and in the general Chinese population. (XLS)

S2 Table Associations between genotypes of p14ARF: rs3814960 and prognosis values of ESCC. OS, overall survival; DFS, disease-free survival; ESCC, esophageal squamous cell carcinoma; HR, hazard ratio; CI, confidence interval. Note: Multivariate analyses in this table were adjusted for patient sex, age, smoking status, drinking status, tumor length, tumor location, differential degree and tumor stage. Adjuvant therapy was not included in the multivariable analysis, because this depends on the tumor stage. *P*c: *P*- value corrected by Benjamini and Hochberg False Discovery Rate correction.
(XLS)

S3 Table Associations between genotypes of Mdm2: rs34886328 and prognosis values of ESCC. OS, overall survival; DFS, disease-free survival; ESCC, esophageal squamous cell carcinoma; HR, hazard ratio; CI, confidence interval; DEL, deletion
Note: Multivariate analyses in this table were adjusted for patient sex, age, smoking status, drinking status, tumor length, tumor location, differential degree and tumor stage. Adjuvant therapy was not included in the multivariable analysis, because this depends on the tumor stage. *P*c: *P*- value corrected by Benjamini and Hochberg False Discovery Rate correction.
(XLS)

S4 Table Associations between genotypes of Mdm2: rs1632248 and prognosis values of ESCC. OS, overall survival; DFS, disease-free survival; ESCC, esophageal squamous cell carcinoma; HR, hazard ratio; CI, confidence interval. Note: Multivariate analyses in this table were adjusted for patient sex, age, smoking status, drinking status, tumor length, tumor location, differential degree and tumor stage. Adjuvant therapy was not included in the multivariable analysis, because this depends on the tumor stage. *P*c: *P*- value corrected by Benjamini and Hochberg False Discovery Rate correction.
(XLS)

S5 Table Associations between genotypes of Mdm2: 937283 and prognosis values of ESCC. OS, overall survival; DFS, disease-free survival; ESCC, esophageal squamous cell carcinoma; HR, hazard ratio; CI, confidence interval. Note: Multivariate analyses in this table were adjusted for patient sex, age, smoking status, drinking status, tumor length, tumor location, differential degree and tumor stage. Adjuvant therapy was not included in the multivariable analysis, because this depends on the tumor stage. *P*c: *P*- value corrected by Benjamini and Hochberg False Discovery Rate correction.
(XLS)

S6 Table Associations between genotypes of p53: rs1050541 and prognosis values of ESCC. OS, overall survival; DFS, disease-free survival; ESCC, esophageal squamous cell carcinoma; HR, hazard ratio; CI, confidence interval. Note: Multivariate analyses in this table were adjusted for patient sex, age, smoking status, drinking status, tumor length, tumor location, differential degree and tumor stage. Adjuvant therapy was not included in the multivariable analysis, because this depends on the tumor stage. *P*c: *P*- value corrected by Benjamini and Hochberg False Discovery Rate correction.
(XLS)

S7 Table Associations between genotypes of p53: rs1042522 and prognosis values of ESCC. OS, overall survival; DFS, disease-free survival; ESCC, esophageal squamous cell carcinoma; HR, hazard ratio; CI, confidence interval. Note: Multivariate analyses in this table were adjusted for patient sex, age, smoking status, drinking status, tumor length, tumor location, differential degree and tumor stage. Adjuvant therapy was not included in the multivariable analysis, because this depends on the tumor stage. *P*c: *P*- value corrected by Benjamini and Hochberg False Discovery Rate correction.
(XLS)

S8 Table Associations between No. of unfavorable genetypes and prognosis values of ESCC. OS, overall survival; DFS, disease-free survival; ESCC, esophageal squamous cell carcinoma; HR, hazard ratio; CI, confidence interval. Note: Multivariate analyses in this table were adjusted for patient sex, age, smoking status, drinking status, tumor length, tumor location, differential degree and tumor stage. Adjuvant therapy was not included in the multivariable analysis, because this depends on the tumor stage.
(XLS)

S9 Table The Strengthening the Reporting of Observational Studies in Epidemiology (STROBE) Statement. (DOC)

S10 Table The raw clinical data. (XLS)
