# Supplementary material for: Genetic Variants in the p14ARF/MDM2/TP53 Pathway Are Associated with the Prognosis of Esophageal Squamous Cell Carcinoma Patients Treated with Radical Resection
Source: PLoS One. 2016 Jul 14;11(7):e0158613. doi: 10.1371/journal.pone.0158613 (PMC4944974; doi:10.1371/journal.pone.0158613)
Supplement: S9 Table — (DOC) [file pone.0158613.s009.doc]

S9 Table. The Strengthening the Reporting of Observational Studies in Epidemiology (STROBE) Statement

|  | Item No. | Recommendation | Page  No. | Relevant text from manuscript |
| --- | --- | --- | --- | --- |
| **Title and abstract** | 1 | (*a*) Indicate the study’s design with a commonly used term in the title or the abstract | 2 | 124 ESCC patients with radical resection were included in this retrospective study and genotyped using the MassArray method. |
|  |  | (*b*) Provide in the abstract an informative and balanced summary of what was done and what was found | 2 | In this study, we explored the association between genetic variants in the p14ARF/MDM2/p53 pathway and prognosis in ESCC patients with radical resection. 124 ESCC patients with radical resection were included in this retrospective study and genotyped using the MassArray method. According to multivariate Cox hazard analysis and multiple testing, the TC/CC genotype of *p14ARF* rs3814960 was shown to be strongly related to a decreased overall survival (OS) (HR = 2.765, 95% CI: 1.330–5.750, *P* = 0.006, *Pc* = 0.030) and disease-free survival (DFS) (HR = 2.449, 95% CI: 1.301–4.608, *P* = 0.005, *Pc* = 0.025). Moreover, patients with the A/− +AA genotype of *MDM2* rs34886328 had a notably increased OS (HR = 0.272, 95% CI: 0.131–0.564, *P* = 4.7×10^−4^, *Pc* = 0.003) and DFS (HR = 0.217, 95% CI: 0.109–0.429, *P* = 1.1×10^−5^, *Pc* = 6.6×10^−5^). We also found that these two SNPs had a cumulative effect on the prognosis of ESCC, with the OS (*P* < 0.001) and DFS (*P* < 0.001) being shortest for patients carrying both of these unfavorable genotypes. In conclusion, genetic variants of the p14ARF/MDM2/p53 pathway are significantly related to OS and DFS, and may therefore be predictors of the prognosis of ESCC after surgery. |
| Introduction | | | |  |
| Background/rationale | 2 | Explain the scientific background and rationale for the investigation being reported | 3-4 | Esophageal cancer (EC) is the fourth most common cancer diagnosed in China [1], and squamous cell carcinomas account for more than 90% of esophageal cancer in high-risk areas such as north-central China, Central Asian countries, and Northern Iran [2]. Despite new developments in early diagnosis and treatment, including surgery, radiation and chemotherapy, prognosis remains poor owing to frequent local recurrence or distant metastasis [3,4]. The overall five-year survival rate of ESCC is still less than 15%, and the clinical variables currently used to predict outcomes are imprecise [5,6]. Thus, the identification of molecular prognostic markers might enable further risk stratification, which could be the first step towards the individualization of treatment strategies [7].  In addition to the patient- and treatment- related factors reported by previous studies, including tumor stage and chemoradiotherapy, plasma values of p53 were recently found to be associated with the prognosis of ESCC [8]. Further, single nucleotide polymorphisms (SNPs) of p53 were found by us to be related to the development of ESCC previously [9]. p53 is a key regulator of the G1/S cell cycle checkpoint [10]. As an important negative regulator of p53, MDM2 inhibits its function by concealing the activation domain of p53 [11,12] and by promoting degradation of p53, most likely through the ubiquitin-proteasome pathway [13,14]. p14ARF can activate the p53 pathway by interacting with and inhibiting the ubiquitin ligase activity of MDM2, preventing the polyubiquitination, nuclear export, and cytoplasmic degradation of p53 [15]. The p14ARF/MDM2/p53 pathway is therefore critical for normal cell cycle progression [16], and abnormalities of the p14ARF/MDM2/p53 pathway are important mechanisms in the development and progression of cancers.  p14ARF, MDM2 and p53 have been shown to present frequent mutations in many tumors [17-19]; However, to the best of our knowledge, no studies have addressed the role of genetic variants of the p14ARF/MDM2/p53 signaling pathway in the prognosis of ESCC. |
| Objectives | 3 | State specific objectives, including any prespecified hypotheses | 4 | We postulated that SNPs in the p14ARF/MDM2/p53 pathway may be associated with the survival and recurrence of ESCC. To verify this hypothesis, we selected six potentially functional SNPs from p14ARF, MDM2 and p53 to discover their potential associations with the OS and DFS of ESCC patients treated with radical resection. |
| Methods | | | |  |
| Study design | 4 | Present key elements of study design early in the paper | 4-6 | We retrospectively analyzed 124 patients treated with esophagectomy at the Department of Thoracic Surgery, Tongji Hospital of Huazhong University of Science and Technology (Wuhan, Hubei Province, China) between March 2010 and December 2012. Patients who had R0 resection for proven ESCCs confirmed by pathologists and received postoperative treatment according to the guidelines of China's Ministry of Health were included. The exclusion criteria included perioperative death and distal metastasis. Clinicopathological information was obtained retrospectively from patient records and authors had access to identifying information during and after data collection. After surgery, patients were followed-up every 4 months for the first 2 years, and every 6 months thereafter. Clinical information and physical examinations were included in each follow-up visit. Routine diagnostic imaging methods were used, including barium meal fluoroscopy and computed tomography, as well as tumor marker assays. The last follow-up was in May 2015.  We collected the patients’ paraffin sections after esophagectomy from the Department of Pathology, Tongji Hospital. Genomic DNA was extracted with [QIAamp DNA FFPE tissue kit](http://www.baidu.com/link?url=0jy-U0XKBPfFjS_I-KCtzWryzDSiTEQALNdHLGOWxtREznFoG25lpzwLtlsgg7WTjE9wQBsoD4-d9Yjp6N2cY_)s (56404; Qiagen, Dusseldorf, Germany) from paraffin sections. Haploview software was used to choose the single nucleotide polymorphisms (SNPs) in the p14ARF/MDM2/p53 pathway. We chose three key genes (p14ARF, MDM2 and p53) along the p14ARF/MDM2/p53 pathway by a candidate gene approach [20]. All of the SNPs had minor allele frequencies of greater than 20% in the Chinese population based on the HapMap HCB data, and all correlated alleles were captured at r2 > 0.8. We only selected SNPs that were located in the 5′ or 3′-UTR gene region, or were found to be associated with the risk of ESCC by our previous reports, for example rs1042522. SNPs in strong linkage disequilibrium with selected SNPs or SNPs that cannot be determined in a well were excluded. Six SNPs were finally selected. For all six SNPs, genotypes were determined using the MassArray system (Sequenom iPLEX assay, San Diego, CA). The sample DNA was amplified by a multiplex PCR reaction, and the PCR products were then used for a locus-specific single-base extension reaction. Finally, the resulting products were desalted and transferred to a 384-element SpectroCHIP array. The alleles were discriminated by mass spectrometry (Sequenom). |
| Setting | 5 | Describe the setting, locations, and relevant dates, including periods of recruitment, exposure, follow-up, and data collection | 4-5 | We retrospectively analyzed 124 patients treated with esophagectomy at the Department of Thoracic Surgery, Tongji Hospital of Huazhong University of Science and Technology (Wuhan, Hubei Province, China) between March 2010 and December 2012. Patients who had R0 resection for proven ESCCs confirmed by pathologists and received postoperative treatment according to the guidelines of China's Ministry of Health were included. The exclusion criteria included perioperative death and distal metastasis. Clinicopathological information was obtained retrospectively from patient records and authors had access to identifying information during and after data collection. After surgery, patients were followed-up every 4 months for the first 2 years, and every 6 months thereafter. Clinical information and physical examinations were included in each follow-up visit. Routine diagnostic imaging methods were used, including barium meal fluoroscopy and computed tomography, as well as tumor marker assays. The last follow-up was in May 2015. |
| Participants | 6 | (*a*) *Cohort study*—Give the eligibility criteria, and the sources and methods of selection of participants. Describe methods of follow-up  *Case-control study*—Give the eligibility criteria, and the sources and methods of case ascertainment and control selection. Give the rationale for the choice of cases and controls  *Cross-sectional study*—Give the eligibility criteria, and the sources and methods of selection of participants | 4-5 | We retrospectively analyzed 124 patients treated with esophagectomy at the Department of Thoracic Surgery, Tongji Hospital of Huazhong University of Science and Technology (Wuhan, Hubei Province, China) between March 2010 and December 2012. Patients who had R0 resection for proven ESCCs confirmed by pathologists and received postoperative treatment according to the guidelines of China's Ministry of Health were included. The exclusion criteria included perioperative death and distal metastasis. Clinicopathological information was obtained retrospectively from patient records and authors had access to identifying information during and after data collection. After surgery, patients were followed-up every 4 months for the first 2 years, and every 6 months thereafter. Clinical information and physical examinations were included in each follow-up visit. Routine diagnostic imaging methods were used, including barium meal fluoroscopy and computed tomography, as well as tumor marker assays. The last follow-up was in May 2015. |
|  |  | (*b*) *Cohort study*—For matched studies, give matching criteria and number of exposed and unexposed  *Case-control study*—For matched studies, give matching criteria and the number of controls per case | / | Our study is not matched study. |
| Variables | 7 | Clearly define all outcomes, exposures, predictors, potential confounders, and effect modifiers. Give diagnostic criteria, if applicable | 6 | The end points for this study were OS and DFS. OS was defined as the length of time (in months) from the date of surgery to the last follow-up, or death from any cause. DFS was defined as the time (in months) after surgery that the patient was tumor-free. |
| Data sources/ measurement | 8* | For each variable of interest, give sources of data and details of methods of assessment (measurement). Describe comparability of assessment methods if there is more than one group | 4-6 | We retrospectively analyzed 124 patients treated with esophagectomy at the Department of Thoracic Surgery, Tongji Hospital of Huazhong University of Science and Technology (Wuhan, Hubei Province, China) between March 2010 and December 2012. Patients who had R0 resection for proven ESCCs confirmed by pathologists and received postoperative treatment according to the guidelines of China's Ministry of Health were included. The exclusion criteria included perioperative death and distal metastasis. Clinicopathological information was obtained retrospectively from patient records and authors had access to identifying information during and after data collection. After surgery, patients were followed-up every 4 months for the first 2 years, and every 6 months thereafter. Clinical information and physical examinations were included in each follow-up visit. Routine diagnostic imaging methods were used, including barium meal fluoroscopy and computed tomography, as well as tumor marker assays. The last follow-up was in May 2015.  We collected the patients’ paraffin sections after esophagectomy from the Department of Pathology, Tongji Hospital. Genomic DNA was extracted with [QIAamp DNA FFPE tissue kit](http://www.baidu.com/link?url=0jy-U0XKBPfFjS_I-KCtzWryzDSiTEQALNdHLGOWxtREznFoG25lpzwLtlsgg7WTjE9wQBsoD4-d9Yjp6N2cY_)s (56404; Qiagen, Dusseldorf, Germany) from paraffin sections. Haploview software was used to choose the single nucleotide polymorphisms (SNPs) in the p14ARF/MDM2/p53 pathway. We chose three key genes (p14ARF, MDM2 and p53) along the p14ARF/MDM2/p53 pathway by a candidate gene approach [20]. All of the SNPs had minor allele frequencies of greater than 20% in the Chinese population based on the HapMap HCB data, and all correlated alleles were captured at r2 > 0.8. We only selected SNPs that were located in the 5′ or 3′-UTR gene region, or were found to be associated with the risk of ESCC by our previous reports, for example rs1042522. SNPs in strong linkage disequilibrium with selected SNPs or SNPs that cannot be determined in a well were excluded. Six SNPs were finally selected. For all six SNPs, genotypes were determined using the MassArray system (Sequenom iPLEX assay, San Diego, CA). The sample DNA was amplified by a multiplex PCR reaction, and the PCR products were then used for a locus-specific single-base extension reaction. Finally, the resulting products were desalted and transferred to a 384-element SpectroCHIP array. The alleles were discriminated by mass spectrometry (Sequenom). |
| Bias | 9 | Describe any efforts to address potential sources of bias | 4 | Patients who had R0 resection for proven ESCCs confirmed by pathologists and received postoperative treatment according to the guidelines of China's Ministry of Health were included. The exclusion criteria included perioperative death and distal metastasis. |
| Study size | 10 | Explain how the study size was arrived at | 4 | We retrospectively analyzed 124 patients treated with esophagectomy at the Department of Thoracic Surgery, Tongji Hospital of Huazhong University of Science and Technology (Wuhan, Hubei Province, China) between March 2010 and December 2012. |

| Quantitative variables | 11 | Explain how quantitative variables were handled in the analyses. If applicable, describe which groupings were chosen and why | 4-6 | After surgery, patients were followed-up every 4 months for the first 2 years, and every 6 months thereafter. Clinical information and physical examinations were included in each follow-up visit. Routine diagnostic imaging methods were used, including barium meal fluoroscopy and computed tomography, as well as tumor marker assays. The last follow-up was in May 2015.  We collected the patients’ paraffin sections after esophagectomy from the Department of Pathology, Tongji Hospital. Genomic DNA was extracted with [QIAamp DNA FFPE tissue kit](http://www.baidu.com/link?url=0jy-U0XKBPfFjS_I-KCtzWryzDSiTEQALNdHLGOWxtREznFoG25lpzwLtlsgg7WTjE9wQBsoD4-d9Yjp6N2cY_)s (56404; Qiagen, Dusseldorf, Germany) from paraffin sections. Haploview software was used to choose the single nucleotide polymorphisms (SNPs) in the p14ARF/MDM2/p53 pathway. We chose three key genes (p14ARF, MDM2 and p53) along the p14ARF/MDM2/p53 pathway by a candidate gene approach [20]. All of the SNPs had minor allele frequencies of greater than 20% in the Chinese population based on the HapMap HCB data, and all correlated alleles were captured at r2 > 0.8. We only selected SNPs that were located in the 5′ or 3′-UTR gene region, or were found to be associated with the risk of ESCC by our previous reports, for example rs1042522. SNPs in strong linkage disequilibrium with selected SNPs or SNPs that cannot be determined in a well were excluded. Six SNPs were finally selected. For all six SNPs, genotypes were determined using the MassArray system (Sequenom iPLEX assay, San Diego, CA). The sample DNA was amplified by a multiplex PCR reaction, and the PCR products were then used for a locus-specific single-base extension reaction. Finally, the resulting products were desalted and transferred to a 384-element SpectroCHIP array. The alleles were discriminated by mass spectrometry (Sequenom). |
| --- | --- | --- | --- | --- |
| Statistical methods | 12 | (*a*) Describe all statistical methods, including those used to control for confounding | 6 | SPSS 16.0 statistical software (SPSS Inc., Chicago, IL) was used for the statistical analysis. Patients were divided into groups according to their genotypes, and Cox proportional hazard analysis was applied to estimate the hazard ratio (HR) and 95% confidence intervals (CIs) of all possible prognostic factors. Moreover, multivariate Cox regression analysis was used for the adjustment of covariates. The influences of the genotypes on the prognosis were assessed by Kaplan–Meier analysis and compared with log-rank tests. For genotype analysis, P-values were corrected by the Benjamini and Hochberg False Discovery Rate correction. |
| (*b*) Describe any methods used to examine subgroups and interactions | / | None. |
| (*c*) Explain how missing data were addressed | / | None. |
| (*d*) *Cohort study*—If applicable, explain how loss to follow-up was addressed  *Case-control study*—If applicable, explain how matching of cases and controls was addressed  *Cross-sectional study*—If applicable, describe analytical methods taking account of sampling strategy | / | None. |
| (*e*) Describe any sensitivity analyses | / | None. |
| Results | | | | |
| Participants | 13* | (a) Report numbers of individuals at each stage of study—eg numbers potentially eligible, examined for eligibility, confirmed eligible, included in the study, completing follow-up, and analysed | 7 | A total of 124 patients were included in this study, consisting of 96 males and 28 females. Their characteristics are listed in Table [1](http://onlinelibrary.wiley.com/enhanced/doi/10.1002/cam4.564#cam4564-tbl-0001). The median age of all patients was 58 years (range: 40–76 years); 61.3% smoked tobacco, 54.0% drank alcohol, 16.9% had stage III disease, and 42 (33.9%) received adjuvant therapy. |
| (b) Give reasons for non-participation at each stage | / | None. |
| (c) Consider use of a flow diagram | / | None. |
| Descriptive data | 14* | (a) Give characteristics of study participants (eg demographic, clinical, social) and information on exposures and potential confounders | 7 | A total of 124 patients were included in this study, consisting of 96 males and 28 females. Their characteristics are listed in Table [1](http://onlinelibrary.wiley.com/enhanced/doi/10.1002/cam4.564#cam4564-tbl-0001). The median age of all patients was 58 years (range: 40–76 years); 61.3% smoked tobacco, 54.0% drank alcohol, 16.9% had stage III disease, and 42 (33.9%) received adjuvant therapy. |
| (b) Indicate number of participants with missing data for each variable of interest | / | None. |
| (c) *Cohort study*—Summarise follow-up time (eg, average and total amount) | 9 | At a median follow-up interval of 37 months (range: 3–61 months), a total of 41 (33.1%) patients had died and 50 (40.3%) patients had developed tumor recurrences. |
| Outcome data | 15* | *Cohort study*—Report numbers of outcome events or summary measures over time | 7,9 | A total of 124 patients were included in this study, consisting of 96 males and 28 females. Their characteristics are listed in Table [1](http://onlinelibrary.wiley.com/enhanced/doi/10.1002/cam4.564#cam4564-tbl-0001). The median age of all patients was 58 years (range: 40–76 years); 61.3% smoked tobacco, 54.0% drank alcohol, 16.9% had stage III disease, and 42 (33.9%) received adjuvant therapy.  At a median follow-up interval of 37 months (range: 3–61 months), a total of 41 (33.1%) patients had died and 50 (40.3%) patients had developed tumor recurrences. The median OS was 37 months, and the median DFS was 35 months. While in patients who had developed tumor recurrences, the median OS was 20 months, and the median DFS was 13.5 months. |
| *Case-control study—*Report numbers in each exposure category, or summary measures of exposure | / | None. |
| *Cross-sectional study—*Report numbers of outcome events or summary measures | / | None. |
| Main results | 16 | (*a*) Give unadjusted estimates and, if applicable, confounder-adjusted estimates and their precision (eg, 95% confidence interval). Make clear which confounders were adjusted for and why they were included | 9-10 | We found that two SNPs (p14ARF rs3814960 and MDM2 rs34886328) were significantly associated with the prognosis of ESCC (Fig [1](http://onlinelibrary.wiley.com/enhanced/doi/10.1002/cam4.564#cam4564-fig-0002)), determined by the Kaplan–Meier method. Patients with the TC/CC genotype of p14ARF rs3814960 had significantly decreased OS (P = 0.001) and DFS (P = 0.001), while patients with the A/− +AA genotype of MDM2 rs34886328 had significantly increased OS (P = 0.0004) and DFS (P = 0.0001). The other four SNPs were not found to be related to the OS or DFS of ESCC by the Kaplan–Meier method.  Furthermore, multiple Cox proportional hazard analyses with adjustments for patient sex, age, smoking status, drinking status, tumor length, tumor location, differential degree and tumor stage revealed that the TC/CC genotype of p14ARF rs3814960 was strongly related to a decreased OS (HR = 2.765, 95% CI: 1.330–5.750, P = 0.006, Pc = 0.030) and DFS (HR = 2.449, 95% CI: 1.301–4.608, P = 0.005, Pc = 0.025). Moreover, patients with the A/− +AA genotype of MDM2 rs34886328 had a notably increased OS (HR = 0.272, 95% CI: 0.131–0.564, P = 4.7×10^−4^, Pc = 0.003) and DFS (HR = 0.217, 95% CI: 0.109–0.429, P = 1.1×10^−5^, Pc = 6.6×10^−5^) (Table [2](http://onlinelibrary.wiley.com/enhanced/doi/10.1002/cam4.564#cam4564-tbl-0003)). Similar analyses of the other four SNPs showed no associations between any genotype and OS or DFS. |
| (*b*) Report category boundaries when continuous variables were categorized | / | None. |
| (*c*) If relevant, consider translating estimates of relative risk into absolute risk for a meaningful time period | 10 | Furthermore, multiple Cox proportional hazard analyses with adjustments for patient sex, age, smoking status, drinking status, tumor length, tumor location, differential degree and tumor stage revealed that the TC/CC genotype of p14ARF rs3814960 was strongly related to a decreased OS (HR = 2.765, 95% CI: 1.330–5.750, P = 0.006, Pc = 0.030) and DFS (HR = 2.449, 95% CI: 1.301–4.608, P = 0.005, Pc = 0.025). Moreover, patients with the A/− +AA genotype of MDM2 rs34886328 had a notably increased OS (HR = 0.272, 95% CI: 0.131–0.564, P = 4.7×10^−4^, Pc = 0.003) and DFS (HR = 0.217, 95% CI: 0.109–0.429, P = 1.1×10^−5^, Pc = 6.6×10^−5^) (Table [2](http://onlinelibrary.wiley.com/enhanced/doi/10.1002/cam4.564#cam4564-tbl-0003)). Similar analyses of the other four SNPs showed no associations between any genotype and OS or DFS. |

| Other analyses | 17 | Report other analyses done—eg analyses of subgroups and interactions, and sensitivity analyses | / | None. |
| --- | --- | --- | --- | --- |
| Discussion | | | | |
| Key results | 18 | Summarise key results with reference to study objectives | 14 | The current study evaluated genetic variants of *p14ARF*, *MDM2* and *p53* to discover their potential associations with the prognosis of ESCC patients who underwent radical resection. Among them, two SNPs, *p14ARF* rs3814960 and *MDM2* rs34886328, were found to be significantly associated with the OS and the DFS of ESCC. The TC/CC genotype of *p14ARF* rs3814960 was strongly related to a decreased OS and DFS. Moreover, patients with the A/− +AA genotype of *MDM2* rs34886328 had a notably increased OS and DFS. Furthermore, we found that these SNPs had a cumulative effect on the prognosis of ESCC, with the OS and DFS being shortest for patients carrying both of these unfavorable genotypes. |
| Limitations | 19 | Discuss limitations of the study, taking into account sources of potential bias or imprecision. Discuss both direction and magnitude of any potential bias | 16 | In China few patients received chemoradiotherapy before the surgery according to NCCN guidelines. For eliminating the influence of substandard treatment to prognosis, we excluded patients who did not received postoperative treatment according to the guidelines of China's Ministry of Health, which is the reason why only 16.9% patients had stage III disease and only 40.3% patients had developed tumor recurrences in our study. In addition, because of the retrospective nature of this study, and the relatively small population, our findings need to be confirmed by larger, multicenter, prospective studies. Due to the substantial ethnic variation in SNP frequencies, our results, which were demonstrated in a Han Chinese population, should be validated in different ethnic backgrounds. Moreover, rs3814960 and rs34886328 warrant further investigation to identify the causative SNPs and their molecular mechanisms. Furthermore, it is well-known that the p14ARF/MDM2/p53 pathway is one of the most important signaling pathways in cancer development, so we need to explore the potential role of this pathway in the progression of ESCC, which could provide novel insight into the treatment of ESCC. We will also carry out further research to discover the impact of SNPs in the p14ARF/MDM2/p53 pathway on cancer progression in our cohort. |
| Interpretation | 20 | Give a cautious overall interpretation of results considering objectives, limitations, multiplicity of analyses, results from similar studies, and other relevant evidence | 15 | The progression of cancer is a complex process involving environmental, genetic, treatment-related and other factors. The mechanisms of the relevance of the p14ARF/MDM2/p53 pathway to the prognosis of ESCC is not clear. The failure of cell cycle control is a fundamental step in ESCC onset and progression. Key regulation of the G1/S cell cycle checkpoint is provided by p53, together with MDM2 and p14ARF. The N-terminus of MDM2 binds to the transactivation domain of p53 and inhibits its transcriptional activity, and MDM2 also regulates the p53 protein level. p53 is targeted for nuclear export and degradation in the cytoplasm through the ubiquitin-proteasome system, where MDM2 functions as an E3 ubiquitin ligase [25,26]. An autoregulatory loop occurs between p53 and MDM2, where high p53 transactivation is opposed by MDM2 upregulation [27]. p14ARF also takes part in this loop, and is located in the nucleolus, where it sequesters MDM2, inactivates it, and allows stabilization of p53 [28]. This is regulated by p53 in an autoregulatory feedback loop [29]. Generally speaking, the above evidence suggests that the p14ARF/MDM2/p53 pathway is a vital regulator in tumor development and progression, indicating the biological plausibility of the relevance of the p14ARF/MDM2/p53 pathway to the prognosis of ESCC, as shown by our research. |
| Generalisability | 21 | Discuss the generalisability (external validity) of the study results | 14-15 | Although the impact of p53 rs1042522 and MDM2 rs937283 have been extensively studied in relation to the development and progression of many malignances, the results are still inconsistent in esophageal cancer [21-24]. Our previous study showed that the frequency of p53 rs1042522 C/C in ESCC patients was 27.7%, and the risk of ESCC significantly decreased among subjects with the p53 C/C genotype [9]. In the present study, however, the frequency of the p53 rs1042522 C/C genotype in ESCC patients was 29.0%, and we were unable to demonstrate an association between p53 rs1042522 and the prognosis of ESCC. In addition, the present study showed a higher frequency of the MDM2 rs937283 A/A genotype in ESCC patients than that shown by our previous research (62.1% vs. 53.1%). This result may be attributed to the different DNA sources in these studies (peripheral blood vs. ESCC tissue). |
| Other information | |  | | |
| Funding | 22 | Give the source of funding and the role of the funders for the present study and, if applicable, for the original study on which the present article is based | / | This work was supported by the Merck Serono Oncology Rearch Fund of Chinese Society of Clinical Oncology (Y-MT2014-001).  Funders did not participatie into our research. |

*Give information separately for cases and controls in case-control studies and, if applicable, for exposed and unexposed groups in cohort and cross-sectional studies.

**Note:** An Explanation and Elaboration article discusses each checklist item and gives methodological background and published examples of transparent reporting. The STROBE checklist is best used in conjunction with this article (freely available on the Web sites of PLoS Medicine at http://www.plosmedicine.org/, Annals of Internal Medicine at http://www.annals.org/, and Epidemiology at http://www.epidem.com/). Information on the STROBE Initiative is available at www.strobe-statement.org.
